# Factors influencing the happiness of male correctional officers: A cross-sectional study in South Korea

**DOI:** 10.1371/journal.pone.0308171

**Published:** 2024-08-08

**Authors:** Hyun-Ok Jung, Seung-Woo Han

**Affiliations:** 1 College of Nursing, The Research Institute of Nursing Science, Daegu Catholic University, Daegu, South Korea; 2 Department of Nursing, Kwangju Women’s University, Gwangsan-gu, Gwangju, South Korea; Universitas Airlangga, INDONESIA

## Abstract

**Purpose:**

This study is a descriptive research study using a self-reported survey method to determine the impact of correctional officers’ job satisfaction, leisure satisfaction, and family strengths on their happiness.

**Methods:**

This study targeted 269 male correctional officers working in correctional facilities established in P and S cities. The tools used in this study were job satisfaction, leisure satisfaction, and family strengths and happiness. The collected data were analyzed using the SPSS/WIN 22.0 program according to the research purpose. Data analysis included descriptive statistics, analysis of differences in happiness according to demographic characteristics, correlations between variables, and finally, analysis of factors affecting happiness through multiple linear regression.

**Results:**

As a result of this study, job satisfaction(β = 0.081, *p* = 0.036), leisure satisfaction(β = 0.078, *p* = 0.001), and family strengths(β = 0.081, *p* = 0.001) were found to be factors affecting the happiness of male correctional officers, and the explanatory power of these variables was found to be 26.0%.

**Conclusion:**

Based on the results of this study, it is necessary to identify factors that affect the happiness of male correctional officers and provide various counseling and program interventions that can contribute to improving happiness.

## Introduction

As of December 31, 2022, South Korea operated 54 correctional facilities, which employed a total of 16,808 correctional officers, of whom 96.4% work directly with inmates. These officers are increasingly using mental health programs due to the intense job stress associated with the combined responsibilities of inmate management and supervision, rehabilitation and reform. Additional stress factors include overcrowded facilities, shift work, witnessing suicide (attempts), and experiencing assaults by inmates. Analysis of mental health program usage among Korean correctional officers reveals a dramatic increase of 282.18% in the last five years, growing from 1,156 users in 2017 to 4,418 in 2022 [1]. Moreover, correctional officers rate their job satisfaction relatively low, with an overall score of 2.4 out of 5 in job happiness measures, which includes 2.5 for significance in correctional work, 2.9 for pleasure in the work environment, and 2.6 for utilization of abilities and skills, indicating a perception of their occupation as one of the least happy [2].

Happiness is a state of contentment and joy experienced in everyday life, not realized by elimination of negative emotions, but rather by discovery and development of personal strengths, thereby fostering positive emotions in life’s experiences [3]. Recognizing job satisfaction as a key element of workplace quality is one of the foundational steps to increasing personal happiness [4]. Job satisfaction is an evaluative state that represents satisfaction and positive emotions concerning one’s job [5]. Job Satisfaction is a critical requirement for an individual to achieve success, happiness, and productivity. It is particularly essential for correctional officers to enable them to function effectively within correctional facilities [6]. When correctional officers have high job satisfaction, it leads to a sense of happiness at work, increases motivation, interest in their role, and enables them to find meaning and purpose in their work. It also fosters positive relationships with colleagues and dedication to their duties [7].

Leisure satisfaction also influences of individual happiness [8–10]. Leisure activities are voluntary engagement in specific activities during one’s free time, with the purpose of deriving pleasure or happiness [11]. Leisure satisfaction refers to an individual’s positive assessment of their engagement in leisure activities [12]. Engaging in leisure activities helps individuals to build social relationships and acquire new skills and knowledge, so the individual’s likelihood of becoming specialized in these activities increases [10, 13]. As individuals’ leisure activities become increasingly specialized, they experience enhanced self-expression and joy, and develop methods and skills for stress relief, consequently increasing their [9, 10]. Leisure activities can have a positive influence on job satisfaction and efficiency, especially when individuals work in high-stress job environments. The activities help alleviate job stress and increase happiness by enabling individuals to gain valuable experiences in life [13, 14].

Happiness of an individual is intrinsically linked to the happiness of their family. In cultures like South Korea, within the broader Asian context, family happiness is often given more importance than individual happiness. This tradition is due to complex social norms that emphasize kinship relationships and a higher degree of prosocial behavior towards family members compared to other groups [15]. For family happiness, maintenance of harmony, peace, and balance among family members is essential [16]. Family strengths lie in the potential to increase mutual understanding and interaction among family members, to increase sharing of common values and beliefs, and to improve individual capabilities within the family dynamic [17]. Correctional officials often encounter challenges at work due to conflicts between their workplace responsibilities and family life. Such conflicts between job duties and family obligations typically emerge when negative job aspects, like role overload, shift work, and excessively authoritative attitudes, seep into family dynamics [18]. Thus, for correctional officers to achieve personal happiness, they must have support in fostering family strengths.

A review of previous research reveals a lack of quantitative or qualitative studies specifically investigating the happiness of correctional officers. The “bottom-up spillover” theory posits that happiness is directly influenced by the fulfillment of satisfaction in foundational areas such as occupation, leisure activities, family, and income, which in turn affect the broader concept of happiness [19]. Working in correctional facilities entails managing and supervising inmates who have committed crimes, so it is both a rewarding and challenging occupation. If the happiness of individual correctional officers is continuously prioritized, it could increase the efficiency of achieving key correctional goals, such as the successful reintegration of offenders and the prevention of reoffending. Thus, this study focuses on understanding how job satisfaction, leisure satisfaction, and family strengths affect the happiness of correctional officers, aligned with the bottom-up spillover theory of happiness. The findings are intended to serve as foundational data for the development of programs aimed at increasing the happiness of correctional officers and promoting both their physical and their mental health.

## Materials and methods

### Research design

This study used a descriptive survey approach using a self-reported survey method. The primary aim was to investigate how job satisfaction, leisure satisfaction, and family strengths affect the happiness of correctional officers.

### Study subjects

They were male correctional officers, selected by random sampling. The sample size was calculated using the G*Power 3.1 program to ensure adequate power for the study. The criteria included a significance level (α) of 0.05, power (1-β) of 0.95, an effect size of 0.10, and the input of 11 independent variables, yielding a required convenience sample size of 262. Considering the non-return rate of questionnaires and the typical10% drop-out rate due to insincere responses, 290 questionnaires were distributed. After excluding 21 of these due to non-response or insincerity, the final sample size was 269.

### Research tools

#### Job satisfaction

This study used the Korea Minnesota Satisfaction Questionnaire (K-MSQ), which was adapted by Park [20] from the Minnesota Satisfaction Questionnaire (MSQ) [21]. This adaptation was validated for both reliability and validity. The K-MSQ comprises three subscales: extrinsic satisfaction, intrinsic satisfaction, and general satisfaction, totaling 20 items. Each item is scored on a 5-point Likert scale from 1 (very dissatisfied) to 5 (very satisfied), with high scores indicating high satisfaction. In this study, the Cronbach’s alpha for the scale was 0.95.

#### Leisure satisfaction

This study used the Leisure Satisfaction Scale (LSS), developed by [22] and adapted [23]. The scale includes six subscales: psychological, educational, social, relaxation, physical, and aesthetic satisfaction, encompassing a total of 24 items. Each item is rated on a five-point Likert scale, ranging from 1 (not at all) to 5 (very much so), with high scores indicating high leisure satisfaction. In this study, the Cronbach’s alpha for the scale was 0.95.

### Family strengths

This study used the Korea Family Strengths Scale (KFSS-II), developed by Yoo et al. [17] The scale consists of five sub-dimensions: family resilience, mutual respect and acceptance, qualitative bonding, economic stability and cooperation, and family culture and social participation, with a total of 22 items. Each item is rated on a five-point Likert scale, ranging from 1 (not at all) to 5 (very much so), with a high score indicates high family strengths. In this study, the Cronbach’s alpha for the scale was 0.95.

### Happiness

For this study, the shortened Korean version of the happiness scale developed by Suh and Koo [24] was used. This scale consists of nine items encompassing three sub-factors that are central to the experience of happiness: life satisfaction, positive emotions, and negative emotions. Life satisfaction assesses the level of satisfaction with personal aspects, interpersonal relationships, and group memberships. Positive and negative emotions quantify the extent of emotions that are closely associated with happiness. Negative items were reverse-scored. Each item is scored on a seven-point Likert scale, ranging from 1 (not at all) to 7 (very much so), with higher scores indicating greater levels of happiness. In this study, the Cronbach’s alpha for the scale was 0.81.

### Data collection

The data were collected from February 13 to March 10, 2024, targeting correctional officers employed at correctional facilities in cities P and S. The researchers personally visited these facilities, explained the purpose and method of the study to the psychotherapists there, and secured permission and cooperation for the research. Furthermore, the researchers engaged directly with potential participants who met the selection criteria, explained the purpose of the study and the method for responding to the questionnaire to solicit cooperation in data collection. The survey took approximately 20 minutes to complete, and participants who finished the survey received a small reward of appreciation.

### Data analysis

The collected data were analyzed using the SPSS/WIN 22.0 program according to the research objectives. The distribution of the general characteristics of subjects was analyzed using frequency, percentage, mean, and standard deviation. The subjects’ job satisfaction, leisure satisfaction, family strengths, and happiness were also analyzed in terms of mean and standard deviation. Differences in happiness based on the subjects’ general characteristics were analyzed using independent t-tests and one-way ANOVA, with post-hoc analysis conducted using the Scheffé test. The pairwise correlations of job satisfaction, leisure satisfaction, family strengths, and happiness were quantified using Pearson’s correlation coefficients. The factors that influence the happiness of correctional officers were examined using multiple linear regression analysis.

### Ethical considerations

This study was approved by the D University Institutional Review Board (IRB NO. CUIRB-2023-0060). The survey questionnaire incorporated detailed explanations about the necessity and purpose of the study, research method, estimated time commitment, the possibility of withdrawal during the study, voluntary participation, potential benefits and disadvantages of participating, as well as the management and confidentiality of personal information. Contact information for the researchers was also provided for any inquiries related to the study. The survey was administered only to those who understood and consented to these terms, and their responses were used for data analysis. the participants were assured that survey results would be quantified, processed, and used exclusively for the purposes of this research, that their anonymity would be preserved and that they could opt out of the survey at any point without any negative consequences. Following these explanations, written consent was obtained from each participants. They then individually completed the questionnaires, which were subsequently collected directly by the research team.

## Results

### General characteristics of subjects

The results of this study regarding happiness scores based on demographic characteristics are as follows (Table 1). Each of the following comparisons considers the group being presented.

**Table 1:** General characteristics of subjects (*N* = 269).

Variable	N (%)	M (SD)	T or F	*p*
Age				
20s	29 (10.8)	4.51 (0.76)	2.061	0.106
30s	95 (35.3)	4.15 (0.65)		
40s	86 (32.0)	4.34 (0.85)		
50s	59 (21.9)	4.28 (0.69)		
Marital status				
Single^a^	117 (43.5)	4.22 (0.73)	4.319	0.014 (c < a, b)
Married^b^	150 (55.8)	4.34 (0.73)		
Other^c^	2 (0.7)	2.94 (0.08)		
Number of children				
0	134 (49.8)	4.21 (0.71)	0.998	0.394
1	47 (17.5)	4.29 (0.67)		
2	77 (28.6)	4.39 (0.84)		
≥ 3	11 (4.1)	4.22 (0.70)		
Religious affiliation				
Yes	102 (37.9)	4.19 (0.64)	-1.498	0.135
No	167 (62.1)	4.33 (0.79)		
Years of service				
≤ 10	128 (47.6)	4.22 (0.76)	1.083	0.357
11–20	74 (27.5)	4.30 (0.74)		
21–30	50 (18.6)	4.30 (0.70)		
≥ 31	17 (6.3)	4.56 (0.72)		
Rank				
Grade 9	74 (27.5)	4.22 (0.74)	0.632	0.595
Grade 8	58 (21.6)	4.27 (0.60)		
Grade 7	86 (32.0)	4.26 (0.90)		
Grade 6	51 (19.0)	4.40 (0.61)		
Areas of work				
Security	215 (79.9)	4.26 (0.72)	1.039	0.395
Medical	8 (3.0)	4.18 (1.05)		
Social reintegration	13 (4.8)	4.26 (0.43)		
Vocational training	8 (3.0)	4.75 (0.91)		
Administrative	21 (7.8)	4.23 (0.96)		
Welfare	4 (1.5)	4.75 (0.42)		
Economic status				
Upper	8 (3.0)	4.63 (0.68)	1.608	0.202
Middle	184 (68.4)	4.30 (0.72)		
Lower	77 (28.6)	4.18 (0.80)		

#### Age

The most populous age group was the 40s, with 32.0% (86 subjects). This group also had the highest average happiness score of 4.34 (± 0.85), although the difference was not statistically significant (*p* = 0.106).

#### Marital status

The ’Married’ category was the largest, with 55.8% (150 subjects); it also recorded the highest average happiness score of 4.34 (±0.73). This difference was statistically significant (*p* = 0.014).

#### Number of children

The most common number of children was ’0’, with 49.8% (134 subjects). The highest average happiness score was among those with two children, scoring 4.39 (± 0.84), but this difference was not statistically significant (*p* = 0.394).

#### Religious affiliation

The largest group was those with ’No religion’, with 62.1% (167 subjects); this group also had the highest average happiness score at 4.33 (± 0.79), but the difference was not statistically significant (*p* = 0.135).

#### Years of service

The largest group was those with ’10 years or less’ of service, accounting for 47.6% (128 subjects). The highest average happiness score was among those with ’more than 31 years’ of service, at 4.56 (±0.72), although this difference was not statistically significant (*p* = 0.357).

#### Rank

’Grade 9’ had the highest representation with 27.5% (74 subjects). However, ’Grade 6’ had the highest average happiness score was of 4.40 (±0.61), but this difference was not statistically significant (*p* = 0.595).

#### Area of work

Most of the participants were in the ’Security’ department with 79.9% (215 subjects). The highest average happiness scores were in the ’Welfare’ and ’Vocational Training’ departments, both scoring 4.75 (±0.91, 0.42), but the differences were not statistically significant (*p* = 0.395).

#### Economic status

Most of the participants were in the ’middle’ category, with 68.4% (184 subjects). However, the ‘upper’ category had the highest average happiness of 4.63 (±0.68), although the difference was not statistically significant (*p* = 0.202).

### Levels of job satisfaction, leisure satisfaction, family strengths, and happiness among subjects

The average job satisfaction score among the subjects was 3.30 (±0.56). The average leisure satisfaction was 3.56 (±0.62), and average family strengths was 3.61 (±0.62). Lastly, the average happiness score was 4.28 (±0.74) (Table 2).

**Table 2:** Levels of job satisfaction, leisure satisfaction, family strengths, and happiness among subjects (*N* = 269).

Category	M (SD)	Min	Max
Job satisfaction	3.30 (0.56)	1.00	5.00
Leisure satisfaction	3.56 (0.62)	1.50	5.00
Family strengths	3.61 (0.62)	1.91	5.00
Happiness	4.28 (0.74)	2.00	7.00

### Correlations among job satisfaction, leisure satisfaction, family strengths, and happiness

This study analyzed the correlations among job satisfaction, leisure satisfaction, family strengths, and happiness among the subjects. The analysis revealed significant positive correlations between happiness and job satisfaction (r = 0.336, *p* < 0.001), leisure satisfaction (r = 0.433, *p* < 0.001), and family strengths (r = 0.459, *p* < 0.001) (Table 3).

**Table 3:** Correlations among job satisfaction, leisure satisfaction, family strengths, and happiness (*N* = 269).

Category	Job satisfaction	Leisure satisfaction	Family strengths	Happiness
	r (*p)*	r (*p)*	r (*p)*	r (*p)*
Job satisfaction	1			
Leisure satisfaction	0.433 (<0.001)	1		
Family strengths	0.433 (<0.001)	0.554 (<0.001)	1	
Happiness	0.336 (<0.001)	0.433 (<0.001)	0.459 (<0.001)	1

### Factors influencing happiness

In this study, the factors that significantly influenced the happiness of male correctional officers were job satisfaction (β = 0.081, *p* = 0.036), family strengths (β = 0.081, *p* = 0.001), and leisure satisfaction (β = 0.078, *p* = 0.001), in that order. The total explanatory power of these variables was 26.0%. To assess the correlations among these indices, multicollinearity analysis was conducted. The Tolerance Limit in this study ranged between 0.624 and 0.995, which is well above the critical threshold of 0.1. Additionally, the Variance Inflation Factor (VIF), which is considered indicative of multicollinearity if it exceeds 10, was found to be between 1.005 and 1.603. These values suggest that multicollinearity was not a problem in this analysis. The Durbin-Watson statistic was 1.896, which demonstrates that the residuals were independent (Table 4).

**Table 4:** Factors influencing happiness (*N* = 269).

Factors	B	β	SE	t	*p*
(Constant)	1.587	0.289		5.492	0.001
Marital status (ref: Single)					
Married	0.021	0.080	0.014	.260	0.795
Other	-1.127	0.639	-0.094	-1.762	0.079
Job satisfaction	0.171	0.081	0.128	2.106	0.036
Leisure satisfaction	0.266	0.078	0.223	3.392	0.001
Family strengths	0.325	0.081	0.270	4.033	0.001

Durbin-Watson = 1.896, Tolerance Limit = 0.624~0.995, VIF = 1.005~1.603

R^**2**^ = 0.274, Adjusted R^**2**^ = 0.260, F = 19.535, *p* < 0.001

## Discussion

No one would dispute that happiness is the ultimate goal of an individual’s [25]. Grounded in the bottom-up spillover theory of happiness, this study identified job satisfaction, family strengths, and leisure satisfaction as the key factors that influence the happiness of Korean male correctional officers.

This result is consistent with a study of 2,246 teachers in Kalimantan, Indonesia, which found job satisfaction to be a significant factor in increasing happiness [26], and with a study of 494 employees in Spain, which identified job satisfaction is an important predictor of happiness [27].

In Korean correctional institutions in 2022, 1,527 correctional incidents occurred, including suicides, fires, and assaults. Furthermore, detainees made 4,187 complaints to the National Human Rights Commission of Korea, 508 petitions, and 716 criminal charges and accusations against correctional officers. These incidents demonstrate the numerous challenges associated with the duties of correctional officers in South Korea [1].

Job satisfaction is an emotional state of pleasure related to one’s job. Individuals with high job satisfaction possess positive attitudes towards their jobs, whereas those with low job satisfaction tend to have negative attitudes [28]. Positive emotions contribute to increasing an individual’s emotional well-being; they also help to regulate negative emotions [29]. Correctional officers who find their job satisfying and are enthusiastic tend to feel a sense of interest and belonging within their organization. They are more capable than officers who have low job satisfaction at resolving problems with positive rather than negative emotions, and thereby achieving optimal performance; this observation indicate the effect of job satisfaction on happiness [26]. Hence, job satisfaction is an influential factor in happiness. This relationship emphasizes the need to consider job satisfaction when developing happiness-enhancement programs for correctional officers.

The finding that family strengths affect the happiness of Korean male correctional officers is supported by research which shows that family strengths are intimately related to individual happiness. Chinese adults form families through marriage, sharing financial and psychological burdens with their spouses, finding joy in child-rearing, and fostering close relationships with family members. These aspects of family strengths significantly contribute to their happiness [30]. This view is corroborated by research from Hong Kong, which showed that increasing both verbal and non-verbal communication among family members (including direct and indirect methods like phone calls, social media, and emails) strengthens family interaction, cohesion, and adaptability, and thereby increases levels of happiness [31]. Caring for one’s family is considered one of the most fundamental human motivations [32], with the family unit being a pivotal aspect of personal life [33]. Within a framework that emphasizes strong family dynamics, every member holds strengths and potentials to overcome challenges and achieve growth, thereby fostering positive future development and transformation. Individuals nurtured in such healthy family environments are better equipped than those who are not, to care for themselves and establish stable families [17].

South Koreans tend to favor a group-centric rather over an individual-centric orientation. In South Korea, interpersonal relationships tend to be less fluid and open compared to Western cultures, to individuals develop a preference for sustaining long-term relationships with existing families or partners rather than seeking new ones [15, 33]. Specifically for correctional officers, the conflict between workplace responsibilities and family life can lead to problems both at home and at work [18]; this relationship emphasizes the influence of family strengths on happiness of correctional officers. Consequently, family strengths must be included as a component of happiness enhancement programs for correctional officers.

This study’s findings on the influence of leisure satisfaction on the happiness of male correctional officers in Korea concurs with a study of 864 caregivers in Korea, which demonstrated that satisfaction with leisure activities significantly boosts happiness by fostering positive attitudes toward leisure activities and policies [9]. This notion is further supported by a study of 284 Korean adults, which found that individuals who earnestly participate in and devote time and effort to leisure activities gain increased confidence and a sense of achievement, which increase their happiness [34].

Korean correctional officers encounter high levels of job stress due to direct exposure to physical, mental, and emotional violence from inmates. They also face administrative risks such as dealing with petitions, information disclosure requests, and complaints lodged with the National Human Rights Commission, along with legal risks including lawsuits and accusations [1]. Despite these challenges, Korean correctional officers navigate job stress by enduring, mastering inmate management techniques, and regaining their footing, a process that is composed of three stages. During this time, they often find relief from job stress by participating in leisure activities such as exercising, hiking, and traveling [35]. Leisure satisfaction stems from the positive perceptions or emotions that arise from engaging in leisure activities and fulfilling personal needs [22]. Correctional officers probably experience an increase in psychological satisfaction and overall happiness, because these leisure activities enable them to experience positive emotions like joy, and can help in either preventing or alleviating stress [9]. Therefore, the element of leisure satisfaction must be considered when developing programs to increase the happiness of correctional officers.

The limitations of this study are as follows. It focused entirely on male correctional officers, because most of correctional officers are male. Future research should include female correctional officers, and would thus broaden the implications of the analysis. Furthermore, the study was conducted among correctional officers working in facilities located in two specific cities (P and S). Consequently, these findings may not be generalizable to all correctional officers across South Korea. Future studies should consider a nationwide selection of regions. Finally, this study, as a quantitative investigation into the factors influencing the happiness of male correctional officers, may not capture and understand the nuances of happiness even within this specific occupational group. Future studies should incorporate qualitative methods to deepen the insight into the lived experiences of happiness among correctional officers, and to thoroughly explore the essence of happiness and its determinants.

Despite these constraints, this study is significant in its analysis of factors that could affect the mental health of correctional officers, especially within the male-dominated sector of this profession. This study contributes basic data that are essential for developing programs that aim to improve the mental health of correctional officers, and for informing policy recommendations for mental health enhancement. Moreover, this work is distinct from previous research on populations working in high-risk and specialized settings like correctional facilities, which has predominantly focused on factors that detrimentally affect mental health at work, such as burnout and job stress. In contrast, this identified factors such as happiness, that positively affect mental health in the workplace, and sought to identify elements that can positively transform the challenges experienced at work and in everyday life.

## Conclusion

Correctional officers perform a range of physically- and psychologically-demanding tasks within a restricted work environment. The diverse physical and psychological challenges in their work settings reduce their ability to cope with mental health problems and also significantly affect their psychological well-being. This study has revealed that job satisfaction, family strengths, and leisure satisfaction are key factors that influence the happiness of male correctional officers. Consequently, these findings indicate that future research to develop targeted programs and ongoing counseling services should aim to increase psychological well-being, such as happiness, particularly for correctional officers who have a high-risk occupation.

## Supporting information

S1 Dataset This research dataset file. (XLSX)

## References

[1] 2023 Korean Correctional Statistics Yearbook [cited 2024 February 10]. https://www.corrections.go.kr/bbs/corrections/421/573538/artclView.do (In Korean)

[2] CareerExplorer. Are correctional officers happy? 2023 [cited 2024 February 5]. https://www.careerexplorer.com/careers/correctional-officer/satisfaction/

[3] Conway R. Flourish: A new understanding of happiness and well-being-and how to achieve them, by Martin E.P.Seligman. J Posit Psychol. 2012 Dec;7(2):159–161. doi: 10.1080/17439760.2011.614831

[4] Javanmardnejad S, Bandari R, Karimooi MH, Rejeh N, Nia HS, Montazeri A. Happiness, quality of working life, and job satisfaction among nurses working in emergency departments in Iran. Health Qual Life Outcomes. 2021 Apr;19:112. doi: 10.1186/s12955-021-01755-3 33794917 PMC8017644

[5] Judge TA, Hulin CL, Dalal RS. Job satisfaction and job affect. The Oxford handbook of organizational psychology. 2012 July;1:496–525. doi: 10.1093/oxfordhb/9780199928309.001.0001

[6] Lambert EG, Hogan NL. The importance of job satisfaction and organizational commitment in shaping turnover intent: A test of a causal model. Crim Justice Rev. 2009;34(1):96–118. doi: 10.1177/0734016808324230

[7] Kun A, Gadanecz P. Workplace happiness, well-being and their relationship with psychological capital: A study of Hungarian teachers. Curr Psychol. 2022 Jan;41(1):185–199. doi: 10.1007/s12144-019-00550-0

[8] Liu H, Chen X, Zhang H. Leisure satisfaction and happiness: The moderating role of religion. Leis Stud. 2021 Mar;40(2):212–260. doi: 10.1080/02614367.2020.1808051

[9] Yoo J. Attitude toward leisure, satisfaction with leisure policy, and happiness are mediated by satisfaction with leisure activities. Sci Rep. 2022 July;12:11723. doi: 10.1038/s41598-022-16012-w 35810206 PMC9271054

[10] Kim JH, Kil NY, Oitas NA, Hwang S. Exploring the relationships across recreation specialization, leisure satisfaction, and happiness: The case of Korean hikers. Leis Sci. 2023 Apr;(1):1–18. doi: 10.1080/01490400.2023.2197901

[11] Verghese J, LeValley A, Derby C, Kuslansky G, Katz M, Hall C, et al. Leisure activities and the risk of amnestic mild cognitive impairment in the elderly. Neurol. 2006 Mar;66(6):821–827. doi: 10.1212/01.wnl.0000202520.68987.48 16467493 PMC1415273

[12] Ragheb MG, Tate RL. A behavioural model of leisure participation, based on leisure attitude, motivation and satisfaction. Leis Stud. 1993;12(1):61–70. doi: 10.1080/02614369300390051

[13] Duyan M, Gunel L. The effect of leisure satisfaction on job satisfaction: A research on physical education teachers. Int J Appl Exerc Physiol. 2020 Jan;9(12):68–75.

[14] Dallmeyer S, Wicker P, Breuer C. The relationship between leisure time physical activity and job satisfaction: A dynamic panel data approach. J Occup Health. 2023 Dec;65(1):e12382. doi: 10.1002/1348-9585.12382 36627728 PMC9832214

[15] Krys K, Yeung JC, Haas BW, Osch Y, Kosiarczyk A, Kocimska-Zych A, et al. Family first: Evidence of consistency and variation in the value of family versus personal happiness across 49 different cultures. J Cross Cult Psychol. 2023 Mar;54(3):323–339. doi: 10.1177/00220221221134711

[16] Mai H, Le HA. The interdependence of happiness and filial piety within the family: A study in Vietnam. Health Psychol Report. 2023 Oct:17091. doi: 10.5114/hpr/172091 38628278 PMC11016942

[17] Yoo YG, Lee IS, Kim SK, Choi HJ. Development of Korea family strengths scale(KFSS-II). J Kor Home Manag Assoc. 2013 Aug;31(4):113–129. doi: 10.7466/JKHMA.2013.31.4.113 (In Korean)

[18] Vickovic SG, Morrow WJ. Examining the influence of work–family conflict on job stress, job satisfaction, and organizational commitment among correctional officers. Crim Justice Rev. 2019 Jul;45(1):5–25. doi: 10.1177/0734016819863099

[19] Diener E. Subjective well-being. Psychol Bull. 1984;95(3):542–575. doi: 10.1037/0033-2909.95.3.542 6399758

[20] Park SY, Kim JH. Campus life adaptation scale for nursing undergraduates: development and psychometric evaluation. Nurse Educ Today. 2019 Aug;79:56–62. doi: 10.1016/j.nedt.2019.05.014 31103841

[21] Dawis RV, Lofquist LH. Apsychological theory of work adjustment. Minnesota: Univ of Minnesota Press;1984.

[22] Beard JG, Ragheb MG. Measuring leisure satisfaction. J Leis Res. 1980 Feb;12(1):20–33. doi: 10.1080/00222216.1980.11969416

[23] Kim ML, Lee YJ, Hwang SH. Cross-cultural validation test and application of LSS-short form. J Kor Con Assoc. 2010 Nov;10(11):435–445. doi: 10.5392/JKCA.2010.10.11.435 (In Korean)

[24] Suh EK, Koo JS. A concise measure of subjective well-being (COMOSWB): Scale development and validation. Kor J Soc Pers Psychol. 2011 Feb;25(1):95–113. doi: 10.21193/KJSPP.2011.25.1.006 (In Korean)

[25] Tadić M, Bakker AB, Oerlemans WGM. Work happiness among teachers: A day reconstruction study on the role of self-concordance. J Sch Psychol. 2013 Dec;51(6):735–750. doi: 10.1016/j.jsp.2013.07.002 24295146

[26] Hamu FJ, Wea D, Putranti HRD. Determinant spiritual school well-being factors of happiness and job satisfaction for teachers in Central Kalimantan. Jurnal Pijar. 2022 Dec;8(4):1087–1099. doi: 10.33394/jk.v8i4.6195

[27] Merida-Lopez S, Extremera N, Quintana-Orts C, Rey L. In pursuit of job satisfaction and happiness: Testing the interactive contribution of emotion-regulation ability and workplace social support. Scand J Psychol. 2019 Feb;60:59–66. doi: 10.1111/sjop.12483 30240014

[28] Wright TA, Bonett DG. Job satisfaction and psychological well-being as nonadditive predictors of workplace turnove. J Manage. 2007 Apr;33(2):141–160. doi: 10.1177/0149206306297582

[29] Aziz R, Mangestuti R, Wahyuni EN. What Makes the Teacher Happy? Int Conf Recent Innov. 2020 Jan;1:1458–1463. doi: 10.5220/0009929614581463

[30] Xu J, Liu A. Family life and Chinese adults’ happiness across the life span. Chin J Sociol. 2021 Nov;7(4):514–534. doi: 10.1177/2057150X211045484

[31] Wang MP, Chu JTW, Viswanath K, Wan A, Lam T, & Chan SS. Using information and communication technologies for family communication and its association with family well-being in Hong Kong: Family project. J Med Internet Res. 2015 Aug:17(8):e207. doi: 10.2196/jmir.4722 26303434 PMC4642799

[32] Ko A, Pick CM, Kwon JY, Barlev M, Krems JA, Varnum MEW, et al. Family matters: Rethinking the psychology of human social motivation. Perspect Psychol Sci. 2020 Jan;15(1):173–201. doi: 10.1177/1745691619872986 31791196

[33] Krys K, Capaldi CA, Zelenski JM, Park J, Nader M, Kocimska-Zych A, et al. Family well-being is valued more than personal well-being: A four-country study. Curr Psychol. 2021 Apr;40:3332–3343. doi: 10.1007/s12144-019-00249-2

[34] Kim HH, Park IH, Bae JS. Happy campers? The relationships between leisure functioning, serious leisure, and happiness. Soc Behav Pers. 2019 Nov;47(11):e8270. doi: 10.2224/sbp.8270

[35] Jung HO, Kim SK. A grounded theory approach on correctional officers’ adaptation process of job stress. J Kor Acad Community Health Nurs. 2021 Mar;32(2):73–85. doi: 10.12799/jkachn.2021.32.1.73 (In Korean)

